# Predictors of Long-Term Care Utilization by Dutch Hospital Patients aged 65+

**DOI:** 10.1186/1472-6963-10-110

**Published:** 2010-05-06

**Authors:** Albert Wong, Rianne Elderkamp-de Groot, Johan Polder, Job van Exel

**Affiliations:** 1 Department of Statistics and Mathematical Modeling, Expertise Centre for Methodology and Information Services, National Institute for Public Health and the Environment, PO Box 1, 3720 BA, Bilthoven, the Netherlands; 2 Department Tranzo, Faculty of Social and Behavioral Sciences, University of Tilburg, Tilburg, the Netherlands; 3 Centre for Public Health Forecasting, National Institute for Public Health and the Environment, Bilthoven, the Netherlands; 4 Institute of Health Policy and Management (iBMG), Erasmus University Rotterdam, the Netherlands; 5 Institute for Medical Technology Assessment (iMTA), Erasmus University Rotterdam, the Netherlands

## Abstract

**Background:**

Long-term care is often associated with high health care expenditures. In the Netherlands, an ageing population will likely increase the demand for long-term care within the near future. The development of risk profiles will not only be useful for projecting future demand, but also for providing clues that may prevent or delay long-term care utilization. Here, we report our identification of predictors of long-term care utilization in a cohort of hospital patients aged 65+ following their discharge from hospital discharge and who, prior to hospital admission, were living at home.

**Methods:**

The data were obtained from three national databases in the Netherlands: the national hospital discharge register, the long-term care expenses register and the population register. Multinomial logistic regression was applied to determine which variables were the best predictors of long-term care utilization. The model included demographic characteristics and several medical diagnoses. The outcome variables were discharge to home with no formal care (reference category), discharge to home with home care, admission to a nursing home and admission to a home for the elderly.

**Results:**

The study cohort consisted of 262,439 hospitalized patients. A higher age, longer stay in the hospital and absence of a spouse were found to be associated with a higher risk of all three types of long-term care. Individuals with a child had a lower risk of requiring residential care. Cerebrovascular diseases [relative risk ratio (RRR) = 11.5] were the strongest disease predictor of nursing home admission, and fractures of the ankle or lower leg (RRR = 6.1) were strong determinants of admission to a home for the elderly. Lung cancer (RRR = 4.9) was the strongest determinant of discharge to the home with home care.

**Conclusions:**

These results emphasize the impact of age, absence/presence of a spouse and disease on long-term care utilization. In an era of demographic and epidemiological changes, not only will hospital use change, but also the need for long-term care following hospital discharge. The results of this study can be used by policy-makers for planning health care utilization services and anticipating future health care needs.

## Background

In countries all over the world, the health sector faces the challenge of an ageing population. It is expected that the prevalence of chronic diseases will rise, and thus the number of people in need of long-term care. This global development will have a significant effect on health care services in terms of capacity, planning and costs. The World Health Organization (WHO) defines long-term care as 'the system of activities undertaken for persons that are not fully capable of self-care on a long-term basis, by informal caregivers (family and friends) or by formal caregivers (professionals). It encompasses a broad array of services, delivered in homes, in communities or in institutional settings' [1]. The Netherlands provides a wide range of long-term care services and facilities, most of which are completely or almost completely covered by national health insurance plan (Exceptional Medical Expenses Act [2]). These include home care and support services, varying from the very basic to more intensive forms, and two types of residential facilities - homes for the elderly and nursing homes (see Additional file 1, and [2-4]). Nursing homes differ from homes for the elderly in that the care and treatments provided in the former are much more intensive than those provided in the latter, with up to 24-h care being provided for the terminally ill in nursing homes.

In 2005, about 18.5% of the total health care budget in the Netherlands was spent on long-term care provided by nursing homes, homes for the elderly and home care, largely to individuals aged 65+ [5]. In the Netherlands, life expectancy has risen from 70.4 years for men and 72.7 years for women in 1950 to 78.0 and 82.3 years, respectively, in 2007 [6]. Due to the ageing of the post-war 'baby-boomers' and a further predicted increase in life expectancy, a substantial further increase in the proportion of elderly in the general population is anticipated from 2010 onwards.

Given the high costs associated with long-term care and the anticipated growth in the demand for such care in the next two decades, there has been an increasing interest among health professionals and policy-makers for less costly care alternatives. This has resulted in the development of risk profiles of future health care utilization that can be used to provide information on the predictors and probabilities of the types of health care. These profiles are not only useful for projecting future demand for different types of health care, but they also provide some degree of insight into possibilities for preventing and delaying long-term care use in general and the costly admission to residential care facilities in particular [7].

To date, studies on the 65+ population have mainly focussed on determinants of admission to a nursing home. Age has been found to be a strong and consistent predictor of admission to a nursing home [8-15], as has the need for care, with lower physical and mental health status associated with an increased risk of institutionalization [7,9,10,14,16,17]. Depression has also been determined to be an important predictor of admission to a nursing home [18-22]. On the basis of an epidemiological study, Bharucha et al. [23] reported that dementia was the strongest disease predictor of admission to a nursing home, with dementia patients having a nearly fivefold higher risk of requiring nursing home facilities. A number of other studies have reported an association with living alone prior to hospitalization [9,17,24-27]. Although this latter finding is not consistent across studies, it emphasizes the protective role often played by spouses and other relatives and the potential role of these informal caregivers in preventing or delaying the institutionalization of elderly patients. A few studies have found that women are more likely to be admitted to a nursing home than men [14,28,29]. However, this effect may in part be related to two of the above-mentioned determinants, age and living alone, as women have a higher life expectancy and tend to outlive their partner. The traditional division of roles in the household has also been put forward as a possible explanation: according to this argument, women are both more used to and willing to perform household and caring tasks, making it more likely that their male partners will be discharged home (rather than vice versa). Studies addressing ethnicity have reported that being non-white decreases the likelihood of institutionalization [9,12,30-32]. The relationship between socio-economic status and admission to an institution has been found to be rather ambiguous, but some studies have reported a positive association between admission to a care facility and a higher income or level of education [12,13,24].

The majority of studies in this field have only considered admission to a nursing home. In addition, these studies have largely focussed on groups of patients with a specific disease, mostly dementia or stroke. Less is known about predictors of discharge from hospital to other types of long-term care services/facilities, particularly in the context of different diseases.

In the study reported here, we have investigated and compared predictors for the hospital discharge of Dutch patients aged 65+ to alternative types of long-term care - i.e., discharge to home without any formal care, discharge to home with home care, discharge to a nursing home and discharge to a home for the elderly. The health care system in the Netherlands is very comprehensive and therefore allows for a clear view of the whole range of health care needs of hospital patients. Using a target group of patients aged 65+ who were living in their own homes prior to hospital admission, we have applied a regression model to predict the long-term care needs of a new patient with a specific combination of diseases, length of hospital stay and social-demographic characteristics. In times of rapid demographic and epidemiological changes, the results of these analyses can help policy-makers to address future health care needs.

## Methods

### Data

This study had a retrospective design based on individual patient-level data obtained from the database of Statistics Netherlands for 2005. Consent for the data was given by Statistics Netherlands. The dataset comprises three registers, i.e. the national hospital discharge register, the exceptional health care expenses register and the Dutch population register. The hospital discharge register contains administrative patient data on hospital admissions, covering all general and academic hospitals and most specialized hospitals in the Netherlands. It includes date of admission and discharge and medical data, such as diagnoses and treatment; outpatient and ambulatory care are not included. The Dutch population register contains demographic data of all registered inhabitants in the Netherlands, including gender, date of birth, zip code, family relations and date of death. The hospital discharge register records were deterministically linked to population register records by Statistics Netherlands using date of birth, gender and zip code as primary linkage keys. The linkage was of good quality as 87.6% of the records were successfully linked. There is a slight bias towards elderly from a non-Western origin but, overall, the data are considered to be representative [33]. Data on long-term care use were retrieved from the register of exceptional health care expenses, which was linked by Statistics Netherlands to the population register records using the same primary linkage keys as mentioned above. The long-term care register contains information on the starting date and the type and amount of long-term care used. In cases where individuals required more than one type of long-term health care, only the type consumed directly after discharge was considered.

The combined dataset therefore comprised basic demographic characteristics and hospital history (e.g., date of admission and discharge, diagnosis) of all individuals discharged from hospital in the Netherlands in 2005 and, when applicable, data on long-term care consumption after hospital discharge.

### Study population

The target group of our study comprised long-term care users aged 65+ who were living at home prior to their admission to hospital, and not utilizing any kind of formal care at that time. This study population was derived as follows. The main source for the population were the hospitalized patients in 2004 (n = 1,414,142). All individuals in this dataset aged 65 years and older were selected (n = 434,142 individuals). Because we focussed on risk factors for long term care utilization, other than end-of-life care, we also excluded hospitalized patients who died in 2004 or 2005. Within the remaining individuals (n = 323,923), hospitalized patients were identified who lived at home prior to hospitalization and did not use any type of formal care. This group was derived by linking the remaining sample to long-term care data for 2004, and excluding individuals who had consumed long-term care in 2004. The final study group therefore consisted of 262,439 persons (11.5% of the population aged 65+ in the Netherlands). For each individual, exactly one year of hospitalization data was used to determine the number of hospital days and diagnoses. More specifically, for individuals that utilized long-term care in 2005 the study-year was defined as the 365 days preceding the first contact with long-term care in 2005. Since the hospital register also contained admissions from 2005, as well as from 2004, all admissions within this year could be retraced. For individuals that did not utilize long-term care in 2005, the study year was defined by picking a random date in 2005, and taking exactly one year preceding this random date.

### Dependent variables

The response variables were the four possible destinations after discharge from hospital: (1) a nursing home, (2) a home for the elderly, (3) home with home care or (4) or home without formal care. The dependent variables to be modelled were the probabilities of each outcome. Since the patients who died during hospitalization or in the year following hospital admission were excluded from our analysis, the probabilities calculated are conditional on the individual being alive at least 1 year after being admitted to hospital.

### Independent variables from the population register

Several demographic variables were included in the various databases that were linked in this study and used to explain the probability of long-term care utilization. Age and age squared were included as continuous variables, with age squared included because of the anticipated non-linear relationship between age and long-term care utilization [16,34]. Further examination of the data confirmed that these relationships were not of linear but parabolic. Cubic splines for age confirmed this parabolic relationship, but the age and age squared terms were retained as the splines offered nearly identical model predictions over age. Other demographic variables were gender and the presence of a spouse or a child in the patient's household. Interactions between spouse and gender and between spouse and child were also examined.

### Independent variables from the hospital register

Length of stay (LOS) in the hospital was defined as the total number of hospital days, accumulated over exactly one year (see above paragraph 'Study population'), with longer hospital stays regarded as a possible indicator of more severe diseases and complications. As binning the LOS and calculating average probabilities for each outcome suggested a parabolic relationship between LOS and each outcome, length of stay squared was also included in the analysis. We also considered LOS smoothing splines, but this resulted in a nearly identical fit. LOS and LOS squared terms were preferred because of their relatively simpler nature. Medical diagnoses were included, based on the International Shortlist for Hospital Morbidity Tabulation (ISHMT) [35]. Only primary diagnoses were considered. This shortlist was compiled by the Hospital Data Project (HDP) of the European Union Health Monitoring Programme and is aimed at maximizing the statistical comparability of hospital care. In 2005, Eurostat, the Organization for Economic Co-operation and Development (OECD) and the Family of International Classifications (WHO-FIC) adopted this shortlist for data collection and presentation. This shortlist is categorized in 130 groups and 20 chapters. Diagnoses related to pregnancy and childbirth, perinatal conditions and congenital malformations were not relevant to our study population and therefore excluded from the analyses. Groups that included unclassified symptoms and factors influencing health status were also considered to be too broad and also excluded.

### Statistical analysis

A regression model was used to explore the predictors of long-term care utilization after hospital discharge. Since the outcome of interest, the discharge destination, has multiple categories, a multinomial logistic regression model was used [36]. Discharge to home without any formal care was chosen as the reference category.

For the demographic variables, male gender, no spouse present and no child present were chosen as reference categories. For the disease variables, the reference category was the group not having that specific disease. A backwards stepwise regression (including demographic variables) was used to determine which diagnoses needed to be included to simplify the model. The use of all remaining diagnoses (110) simultaneously did not lead to convergence (which suggests the model is overspecified), so it was opted to perform a backwards regression per disease chapter as specified in the ISHMT tabulation. Those diseases with a significant coefficient (α ≤ 0.10) for at least one outcome were included in the final model. A total of 23 diagnoses were used in the final model (referred to as 'core diseases' hereafter). This model was run in Stata 9 with the -mlogit- command.

The results are reported as relative risk ratios (RRR). Interpretation of the RRR is similar to that of the odds ratio, with the exception that it involves a specific outcome as a reference group instead of a group with a negative outcome. In terms of absolute effect of the probabilities, the RRR is hard to interpret. Similar to the simple logistic regression model, the model has a non-linear character that does not allow the researcher to associate a RRR in itself with changes in absolute probabilities. The multinomial logistic model also has the added difficulty of multiple categories, all of which can be considered as competing risks. In some cases this means that while a covariate dummy may have a positive RRR for a certain outcome, the probability of the outcome for the dummy taking on a value of 1 may actually be lower than that when the dummy is zero. This occurs when the probability of another outcome category increases even more, such that the probability of the outcome of interest would fall relative to that other outcome [37]. For this reason, a straightforward interpretation of the RRR is complicated. A marginal (partial) effect can be computed, but this is also not easy to interpret, as this effect depends on the chosen set of values for the covariates in terms of effect sign and effect size. We therefore opted to make multiple model predictions, changing the value of one covariate while holding all other covariates constant [37]. By plotting these model predictions, we were able to perform more comprehensive comparisons.

## Results

### Descriptive analysis

The demographic and clinical characteristics of the study cohort are shown in Table 1. The patient population comprised 262,439 individuals with a mean age of 74.2 years [standard deviation (SD) = 6.4] and an almost equal distribution of males and females. Most of the patients lived only with their spouse. The median length of hospital stay was 4 days. The five most prevalent diseases among the 23 diagnoses included in the model were diabetes mellitus, cerebrovascular diseases, coxarthrosis, chronic obstructive pulmonary disease (COPD) and heart failure. About 72% of the patients included in the study had none of these 23 diseases, but they did have at least one of the remaining 87 diseases (79% of all diseases that were considered for model inclusion). Averaged over the study population, most patients (80.3%) returned home without home care after being discharged from hospital. The others returned home with home care (16.1%) or were admitted to a nursing home (2.5%) or a home for the elderly (1.1%). This distribution of outcomes was found to be distinctly different for those patients with the core diseases, as they had a higher probability of formal care.

**Table 1:** Summary statistics and discharge destination after hospital discharge according to the characteristics of the study population.

Categories			n	Percentage of total sample	No formal care (n = 210,972)	Home Care (n = 42,418)	Home for the elderly (n = 2,918)	Nursing home (n = 6,131)
Age, years (mean = 74.23, SD = 6.39)								
65-74			149,750	57.1%	85.3%	12.9%	0.5%	1.4%
75-84			99,242	37.8%	75.1%	20.2%	1.6%	3.2%
84+			13,447	5.1%	64.7%	23.6%	4.5%	7.2%
						
Sex								
Male			132,326	50.4%	82.8%	14.2%	0.8%	2.2%
Female			130,113	49.6%	77.9%	18.2%	1.5%	2.5%
						
Family situation								
Living Alone			70,596	26.9%	73.4%	20.4%	2.6%	3.6%
Spouse			186,674	71.1%	83.1%	14.4%	1.8%	0.7%
Child			18,604	7.1%	81.2%	0.6%	15.8%	2.5%
						
Hospitalization duration, days (median = 4, SD = 13.79)								
01-10			190,094	72.4%	87.3%	11.2%	0.6%	0.8%
11-20			40,824	15.6%	66.1%	27.7%	2.2%	4.1%
20+			31,521	12.0%	57.1%	31.1%	2.5%	9.3%
						
Diagnoses								
Intestinal, stomach and rectum cancer			5,744	2.2%	49.8%	46.1%	1.6%	2.6%
Lung cancer			2,957	1.1%	45.8%	50.1%	1.3%	2.9%
Uterus cancer			662	0.3%	65.1%	32.0%	1.7%	1.2%
Ovary cancer			414	0.2%	48.1%	47.3%	1.9%	2.7%
Prostate cancer			3,351	1.3%	77.1%	20.2%	0.8%	2.0%
Bladder cancer			2,538	1.0%	74.2%	23.9%	0.6%	1.3%
Diabetes mellitus			10,720	4.1%	68.1%	26.3%	1.5%	4.1%
Dementia			1,064	0.4%	48.9%	26.5%	4.6%	20.0%
Schizophrenia			640	0.2%	52.2%	28.1%	3.8%	15.9%
Alzheimer's disease			240	0.1%	57.1%	23.8%	2.9%	16.3%
Epilepsy			1,087	0.4%	67.3%	22.6%	2.2%	7.8%
Heart Failure			8,688	3.3%	65.0%	29.4%	2.3%	3.3%
Cerebrovascular diseases			9,411	3.6%	61.5%	17.9%	1.4%	19.2%
Chronic obstructive pulmonary disease (COPD)			9,107	3.5%	68.3%	27.6%	1.5%	2.6%
Alcoholic liver disease			153	0.1%	54.3%	34.6%	2.6%	8.5%
Infections of skin			915	0.4%	67.2%	28.4%	1.2%	3.2%
Coxarthrosis			9,220	3.5%	62.3%	29.6%	3.4%	4.7%
Gonarthrosis			6,910	2.6%	70.3%	23.1%	2.1%	4.5%
Glomerular disorders			1,329	0.5%	68.9%	29.0%	1.0%	1.1%
Intracranial injury			1,557	0.6%	72.9%	17.4%	2.2%	7.5%
Fracture of elbow and forearm			1,188	0.5%	67.9%	24.4%	2.5%	5.1%
Fracture of femur			4,327	1.7%	46.2%	29.9%	5.5%	18.4%
Fracture of ankle or lower leg			1,141	0.4%	57.6%	26.7%	4.8%	10.9%
Strictly other diagnoses			190,010	72.4%	86.6%	11.8%	0.7%	0.9%

Sample size n = 262,439. With the exception of the 'n' column, which provides absolute numbers, all other values are given as percentages. The percentages in each column for diagnoses do not add up to 100% because patients can have more than one diagnosis. The percentages given for discharge destination do add up to 100%.

The characteristics of the study population in terms of discharge destinations are summarized in Table 1. We identified a positive association between increasing age and the use of long-term care. The proportion of older adults who were institutionalized was greatest among the group of 85+. Males had a higher probability of being discharged to their homes after hospital admission than females. People living alone or with their children only were more likely to be discharged to a nursing home or homes for the elderly than those who lived with a spouse or with a spouse and children. Patients with prostate cancer had the highest probability of going home without home care (77%). Patients with dementia had the highest probability of a discharge to a nursing home (20%), while patients with a fracture of femur had the highest probability of being discharged to a home for the elderly (5.5%). The latter also had the highest probability of receiving formal care after their hospital discharge (100 - 46.18 = 53.82%).

### Demographics predictors of discharge destination

The results of the multinomial regression model are shown in Table 2. In describing these results, we made a distinction between demographic predictors, hospital care utilization predictors and disease predictors. The presence of a spouse can be seen to lower the probability of all three types of long-term care, with a particularly strong effect observed for residential care (homes for the elderly and nursing homes). Although women were found to be more likely than men to use home care services or go to a home for the elderly after discharge from the hospital, there was no significant difference between the sexes in terms of nursing home care. The sex of the spouse also did not significantly affect the probability of nursing home care. The presence of a child was found to be significant only for residential care, while having both a spouse and a child had no significant effect on the probability of long-term care. Age and age squared terms were both significant, but these were hard to interpret, as mentioned above.

**Table 2:** Relationship between background/disease predictors and discharge destination (relative risk ratios, standard errors and statistical significance).

			Home with home care			Home for the elderly care			Nursing home care		
			
Variable			RRR	SE	Sign.	RRR	SE	Sign.	RRR	SE	Sign.
Demographics											
Age (in years) squared			1.00	0.00	***	1.00	0.00	***	1.00	0.00	***
Age (in years)			1.37	0.02	***	1.55	0.08	***	1.34	0.05	***
Female			1.22	0.03	***	1.14	0.07	*	0.97	0.05	
Presence of a spouse			0.65	0.01	***	0.26	0.02	***	0.48	0.02	***
Female spouse			1.25	0.03	***	1.20	0.10	*	1.12	0.07	
Presence of a child			0.97	0.04		0.64	0.07	***	1.17	0.09	*
Female spouse and child			1.03	0.05		1.10	0.21		0.85	0.09	
									
Hospital care utilization											
Hospital days squared			1.00	0.00	***	1.00	0.00	***	1.00	0.00	***
Hospital days			1.08	0.00	***	1.10	0.00	***	1.12	0.00	***
									
Hospital care diagnoses											
Intestinal, stomach and rectum cancer			3.36	0.10	***	1.72	0.19	***	1.25	0.11	*
Lung cancer			4.89	0.20	***	2.61	0.44	***	2.22	0.27	***
Uterus cancer			2.05	0.18	***	1.65	0.52		0.95	0.35	
Ovary cancer			2.88	0.31	***	1.93	0.71		1.45	0.47	
Prostate cancer			1.60	0.08	***	1.14	0.24		1.15	0.16	
Bladder cancer			1.43	0.07	***	0.61	0.16		0.51	0.10	***
Diabetes mellitus			1.32	0.03	***	1.08	0.09		1.00	0.06	
Dementia			2.08	0.18	***	4.09	0.69	***	7.50	0.83	***
Schizophrenia			1.62	0.16	***	2.23	0.51	***	3.89	0.55	***
Alzheimer's disease			1.20	0.22		1.08	0.46		2.14	0.51	**
Epilepsy			1.42	0.11	***	2.25	0.49	***	1.33	0.18	*
Heart Failure			1.26	0.03	***	1.14	0.09		0.65	0.05	***
Cerebrovascular diseases			1.17	0.04	***	1.32	0.12	**	11.55	0.43	***
COPD			1.29	0.03	***	1.06	0.10		0.73	0.05	***
Alcoholic liver disease			2.63	0.49	***	3.84	2.03	*	4.03	1.38	***
Infections of skin			1.61	0.13	***	0.95	0.30		1.00	0.21	
Coxarthrosis			2.54	0.06	***	5.16	0.34	***	4.93	0.28	***
Gonarthrosis			1.69	0.05	***	2.57	0.23	***	3.89	0.26	***
Glomerular disorders			1.31	0.09	***	0.73	0.21		0.32	0.09	***
Intracranial injury			1.10	0.08		1.53	0.28	*	2.21	0.27	***
Fracture of elbow and forearm			1.86	0.14	***	2.27	0.45	***	2.41	0.38	***
Fracture of femur			2.03	0.08	***	3.83	0.30	***	9.30	0.48	***
Fracture of ankle or lower leg			2.21	0.16	***	6.12	0.92	***	8.18	0.93	***

Log likelihood = -135296.99.

Likelihood Ratio (96) = 48248.56 (p < 0.0001).

Pseudo R^2 ^= 0.1513

**Figure 1:**
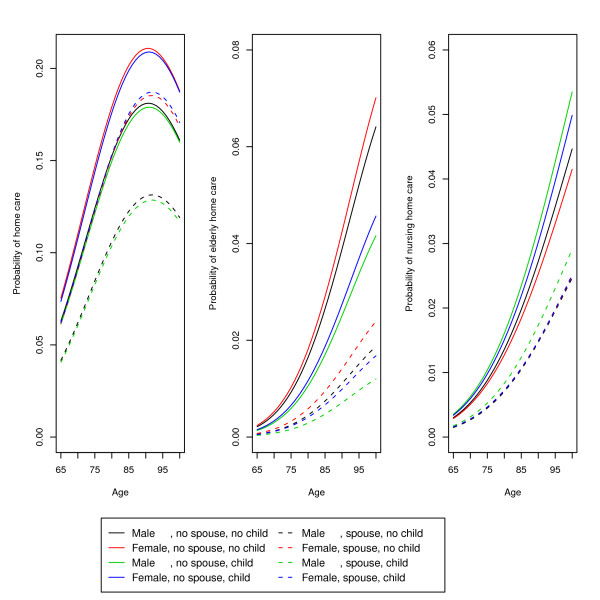
**Predicted probability of discharge for different combinations of gender, presence of spouse and presence of child**. Discharge destinations are home with home care, home for the elderly and a nursing home.

To further clarify the results, we made several model predictions and plotted the results. Figure 1 shows the probability of each type of long-term care for different combinations of gender, presence of a spouse and presence of a child in the household of the patient, plotted against age. In making these predictions, we assumed that the patients had none of the 23 core diseases and had a median length of stay in the hospital. The probability of home care can be seen to be higher than that for either type of residential care. The figure also shows the age-outcome relationship. For residential care, the probability increased with age, with the slope of the line also increasing with age. However, there was a decline in the probability of home care after an age of 90. This figure clearly shows that both men and women with a spouse (dotted lines) had lower probabilities of requiring long-term care than those without a spouse (solid lines). Gender differences were minimal. The presence of a child seems to have had an effect on the use of residential care. Interestingly, those with a child had a higher probability of going home with home care.

### Hospital care utilization

**Figure 2:**
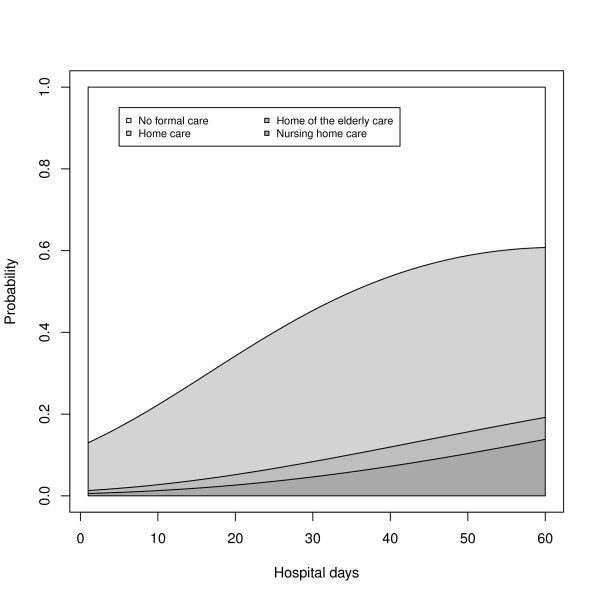
**Predicted cumulative probability of discharge for different lengths of hospitalization**. Discharge destinations are home with no formal care, home with home care, home for the elderly and a nursing home.

Both hospital days RRR terms were significant, but as is the case with age, it was difficult to interpret these coefficients. Figure 2 shows the relationship between the length of stay in the hospital and the distribution of outcomes, based on model predictions for females aged 74 years (mean age) without a spouse or a child in their household and without a model disease. The probability of long-term care increased with increasing stay in the hospital for the first 62 days. This effect was relatively stronger for residential care. After a hospitalization of 62 days, the overall probability of long-term care decreased, while the probability of nursing home and home elderly care combined continued to increase until an age of 87 years. Note that about 99% of all patients had a hospital stay of 62 days or less, which means that model predictions for more than 62+ days have relatively wide confidence intervals (not depicted here).

### Disease predictors of discharge destination

Of all 23 core diseases, all but two had a significant RRR for the probability of going back home with home care. Only five diseases had a non-significant RRR for nursing homes as the discharge destination, and ten diseases had a non-significant RRR for homes for the elderly as discharge destination. The diagnoses with the highest RRR for home care utilization were mainly cancers (lung cancer, intestinal, stomach and rectum cancer and ovary cancer), with alcoholic liver disease and coxarthrosis completing the top five. Of all the cancers examined, only lung cancer and intestinal, stomach and rectum cancer showed a significantly positive RRR for both types of residential care. High RRR for residential care were also found for dementia and alcoholic liver disease and for diseases related to physical functioning (coxarthrosis, gonarthrosis, fracture of femur and fracture of ankle or lower leg). One crucial difference between the forms of residential care was found for cerebrovascular diseases: the RRR was the highest of all diseases for nursing home care, but it was ranked 15th for homes for the elderly.

**Figure 3:**
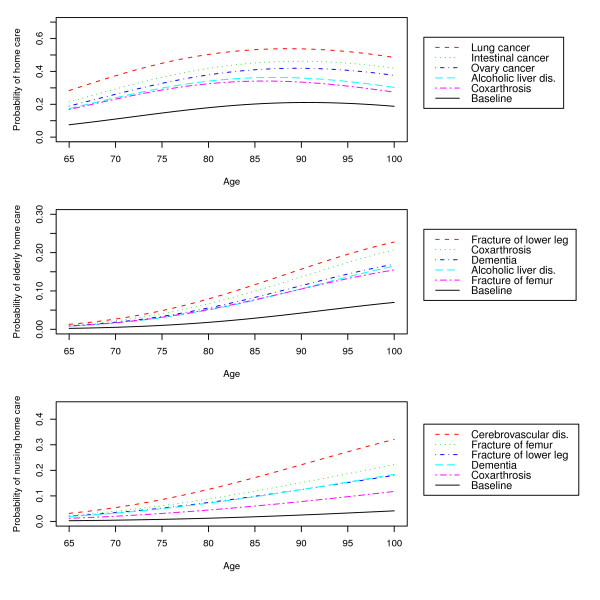
**Five diseases associated with the largest predicted discharge probability for each discharge destination**. Discharge destinations are home with home care, home for the elderly, and a nursing home.

Figure 3 shows the five diseases with the highest probabilities for each outcome. Model predictions were based on female gender, absence of a spouse and child and median LOS. The diagnoses clearly had a larger effect than demographic variables on the probability of formal care. The top five diseases for each outcome category can be seen to correspond with the diseases with the highest RRRs. The differences within these top five diseases are more pronounced for predicting hospital discharge to a nursing home than discharge to a home for the elderly or to the patient's home with home care, with cerebrovascular diseases clearly the most important predictor of nursing home care.

**Figure 4:**
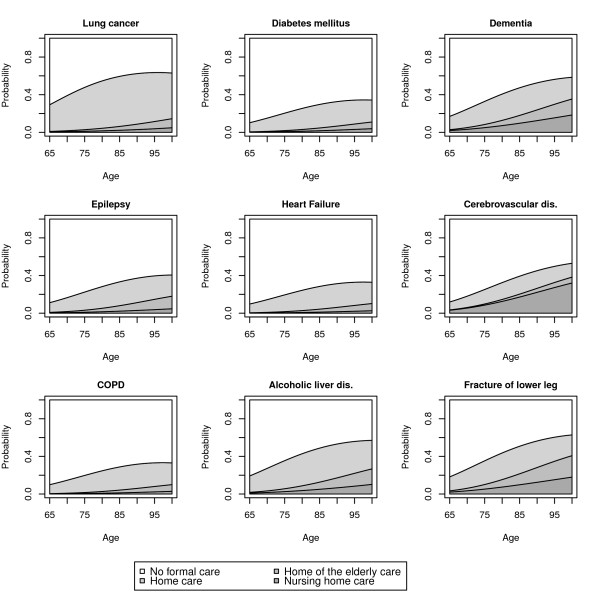
**Predicted cumulative probabilities for discharge destinations associated with nine distinct diseases**. Discharge destinations are home with no formal care, home with home care, home for the elderly and a nursing home.

Figure 4 shows the distribution of outcomes plotted against age for nine diseases. These predictions were based on female gender, absence of a spouse and child and median LOS. All cancers (not depicted) had a similar distribution to that of lung cancer, with a high probability of discharge to the home with home care, while the risk of residential care was comparable to the baseline value (none of the core diseases). On the other end of the spectrum, fracture of the lower leg was associated with a high probability of residential care. Other mobility-related diagnoses (not depicted here) also had comparable patterns. Dementia and cerebrovascular diseases were also good predictors of residential care. Interestingly, heart failure did not lead to nearly as much formal care as cerebrovascular diseases.

## Discussion

The aim of this study was to assess and compare predictors of long-term care utilization by hospital patients aged 65+. By linking data from three national registers, we have been able to show that the large majority of the study population (80.3%) did not use any form of formal long-term care directly after discharge from the hospital. Our results are in agreement with those reported in previous studies [8-15,17,24-27] in showing that higher age, living alone and length of stay in hospital are associated with an increased probability of long-term care utilization.

In general, higher age was associated with an increased risk of long-term care utilization, but above the age of 90 years, the probability of the patient returning home with home care only declined and the probability of residential care increased. This result is very plausible and emphasizes the relevance of using a dataset that includes different types of long-term care.

A longer stay in hospital, within the constraints of the first 62 days, was also associated with a higher risk of formal long-term care.

Living with a spouse or living in the household of a child had a considerable impact on long-term care utilization. The presence of a spouse reduced the risk of all types of long-term care, likely because the spouse was able to provide informal care. Living with a child reduced the risk of admission to a home for the elderly but increased the risk of admission to a nursing home. One possible explanation for this latter effect is that of selection bias. The personal circumstances of an elderly parent(s) who is sharing a household with a child before admission to hospital may differ structurally from those of a parent living alone or with a spouse, such as in terms of frailty and capacity to live independently. Earlier changes in these latter two factors may, in fact, have provided the motivation for the parent to move in with the child in the first place. Although children in such situations may be able to provide informal care, they are likely to have other tasks and responsibilities, such as work and parenting activities. Consequently, there is a critical threshold in their capacity to provide the necessary level of care to the parent: below that level, middle-aged children are able to combine care for their parent with their normal daily activities; beyond that threshold, the burden of care becomes too high and the care of the parent has to be transferred to a facility.

The dynamics of maintaining the patient at home versus institutionalization differ depending on the individuals providing the informal care. Partners tend to be more sensitive to the patient's desire to live at home as long as possible and to feel more responsible (or obliged) to provide the care the patient needs, leading to a longer perseverance time, many times at the expense of their own health [38,39]. We were unable to distinguish between sons and daughters in terms of providing informal care. In contrast, Freedman (1996) showed that having at least one daughter reduced the risk of admission to a home, while the presence of a son did not have any positive effect in this context [25].

Our results are a valuable contribution to existing scientific literature in the field as they provide information on the effect of major diseases in the 65+ population on long-term care utilization following hospital discharge. Based on our data, hospital diagnoses do indeed play a major role in determining the care utilization of the patient following his/her release from hospital. In our model, different diagnoses were the most important predictors for the three types of formal long-term care, with cancers being important predictors of home care, cerebrovascular diseases being important predictors of admission to a nursing home and disability-related diseases, such as fracture of the ankle or of the lower leg, and mental health problems, such as dementia and schizophrenia, being important predictors of admission to a home for the elderly. Changing patterns in hospital morbidity may, therefore, induce a shift in long-term care needs. Policy-makers and health care planners can make use of this information to anticipate on future health care needs.

These findings underline a number of the strengths of this study. Due to the availability of national registers, we were able to include a large number of subjects and a variety of diagnoses and discharge destinations in the analysis. Consequently, we were able to investigate long-term care utilization following discharge from the hospital in a setting with a whole range of long-term care facilities as well as a large variety of disease variables. However, our study also has a number of limitations. First, although the data included information on a large variety of diagnoses, no information was available on the severity of the associated functional and cognitive limitations which, ultimately, can be expected to be stronger predictors of long-term care use. Secondly, this study is largely based on data for a single year (2005). As a consequence we could effectively only observe patients' long-term care utilization up to one year. As such, our conclusions are valid for this single year, and strictly do not regard a subsequent extended period of long term care use. However, given the structure of the Dutch long-term care sector, with comprehensive residential facilities for elderly people, it is common that such long-term decisions are made for the remaining lifetime of the elderly person. Therefore, we expect that the group of elderly with short-term need for extra services is relatively small. We expect that only home care results might be affected by this, and not the residential care. Data covering a longer period would provide more insight into time trends and patterns of long-term care utilization. Regrettably these data were not available. Thirdly, in this study we do not only look at hospitalized patients that utilize long-term care directly after being discharge, but also patients that use long-term care after a certain period following hospitalization. The wider this timeframe is, the weaker the causal link between hospitalization and long-term care becomes. However, the predictors were relatively stable within the observed period of one year. Many of the core diseases are chronic and are therefore unlikely to have become more or less severe in the window. Finally, due to the nature of the data sources used, this study did not take into account a number of other factors that may influence discharge destination, such as supply-side restrictions (e.g., waiting lists), patient preferences and the burden and positive aspects of providing informal care [40-44].

## Conclusions

The results of this study may be relevant to health professionals and policy-makers.

Firstly, our data on the positive influence of the presence of a spouse in terms of home care could have a considerable effect on planning future long-term care use as they provide further evidence that informal care may considerably reduce the need for formal care. In addition, however, this observation is particularly relevant for countries such as the Netherlands in which the gap in life expectancy between men and women is narrowing. It is expected that in the upcoming decades a higher proportion of women will have a spouse, with the result that the need for residential long-term care will show less of an increase relative to conservative estimates. For policy-makers this trend may provide an important incentive to invest in alternative approaches to providing informal care and to enhance and underpin policies that provide support to spousal caregivers.

Secondly, we found that several diagnoses, including cerebrovascular diseases, fractures of the ankle or lower leg, cancers and dementia, are not only important predictors of long-term care utilization but also of specific discharge destinations. Any future change in the prevalence of these diseases will therefore result in changing health care needs, not only within hospitals but also in terms of providing long-term care after discharge. By linking our findings to epidemiological scenarios for the prevalence of these diagnoses, scenarios of future demand for long-term care can be developed and used for capacity and manpower planning of home care, nursing homes and homes for the elderly.

Our figures can also be used for the development of cost-containment measures to limit the financial burden of an ageing population on national budgets. They also highlight the importance of focussing on disease prevention and early intervention as both activities may have a real impact on the number of individuals requiring long-term care.

In conclusion, the ageing of Western societies is a multifaceted phenomenon in which health care needs change much more than has been suggested in traditional scenarios. The need for long-term care following hospital discharge depends heavily on demographic and epidemiological trends. Policy-makers would be advised to anticipate these developments.

## Competing interests

The authors declare that they have no competing interests.

## Authors' contributions

AW and RE jointly formulated the analysis, analysed the data, interpreted the findings and wrote the manuscript draft. JP and JE helped in formulating the analysis and interpreting the findings. They also considerably improved the manuscript by providing suggestions to revise the manuscript. All authors read and approved the final manuscript.

## Pre-publication history

The pre-publication history for this paper can be accessed here:

http://www.biomedcentral.com/1472-6963/10/110/prepub

## Supplementary Material

Additional file 1 **Long-term care in the Netherlands**. Background information on the long-term care system in the Netherlands. Click here for file
