# Supplementary material for: Predictors of Long-Term Care Utilization by Dutch Hospital Patients aged 65+
Source: BMC Health Serv Res. 2010 May 6;10:110. doi: 10.1186/1472-6963-10-110 (PMC2881916; doi:10.1186/1472-6963-10-110)
Supplement: Additional file 1 — Long-term care in the Netherlands. Background information on the long-term care system in the Netherlands. [file 1472-6963-10-110-S1.DOC]

# Long-term care for the elderly in the Netherlands

The Dutch health system comprises three major types of formal care dedicated to the health care needs of the elderly: home care, homes for the elderly and nursing homes [2]. In recent years home care has been restructured into two different categories, but here we describe the situation as it was in 2005, the year of our data collection.

Of all Dutch individuals above the age of 65 years in 2005, about 20% received formal care at home and close to 7% lived in either a nursing home (65,000 people) or a home for the elderly (100,000 people) [3]. Home care largely consisted of personal care and help with daily household activities. The Netherlands has a strong primary care system, and each citizen – elderly or not – can contact his general practitioner in case of sickness or physical/mental complaints. This is also the case for residents of homes for the elderly, which in essence are residential facilities that provide living assistance. Nursing homes provide both personal care and living assistance, with geriatrists and gerontologists responsible for providing integrated medical care. As a general rule, the health status of elderly residents of nursing homes is much poorer than that of the elderly in residential homes.

It is important to note that the *availability* of formal long-term care services is highly regulated [4]. The service capacity within a specific region is directly related to the budget received by local authorities. During recent decades, the national government has promoted an increase in the capacity of community care services and a decrease of the number of beds in institutional care, but compared to many other countries the capacity of residential care in the Netherlands (i.e. homes for the elderly and nursing homes) is still relatively high.

*Access* to formal long-term care services is also strongly regulated. Individuals in need of home care or admission to either a nursing home or a home for the elderly have to apply to a Municipal Committee for a Need Assessment. The applicant may be rejected or, due to a shortage of placements, put on a waiting list. If the request is granted, a (large) share of the costs incurred for formal care utilization is covered by national insurance systems (the Exceptional Medical Expenses Act [2]).
